# Supplementary material for: Identification of Aspergillus niger Aquaporins Involved in Hydrogen Peroxide Signaling
Source: J Fungi (Basel). 2023 Apr 21;9(4):499. doi: 10.3390/jof9040499 (PMC10144872; doi:10.3390/jof9040499)
Supplement: Supplementary file 1 [file jof-09-00499-s001.zip › Supplementary Dataset S1_A niger AQPs sequences. domain identification and classification.pdf]

Identification of *Aspergillus niger* aquaporins involved in hydrogen peroxide signaling

Thanaporn Laothanachareon [1,2,*], Enrique Asin-Garcia [1,3], Rita J.M. Volkers [1], Juan Antonio Tamayo-Ramos [4], Vitor A.P. Martins dos Santos [3] and Peter J. Schaap [1,5,*]

[1] Laboratory of Systems and Synthetic Biology, Wageningen University & Research, 6708 WE Wageningen, The Netherlands; enrique.asingarcia@wur.nl (E.A.-G.); j.m.volkers@nvwa.nl (R.J.V.)

[2] Enzyme Technology Laboratory, Biorefinery and Bioproduct Technology Research Group, National Center for Genetic Engineering and Biotechnology, 113 Thailand Science Park, Khlong Luang, Pathumthani 12120, Thailand

[3] Biomanufacturing and Digital Twins, Wageningen University and Research, 6708 PB Wageningen, The Netherlands; vitor.martinsdossantos@wur.nl

[4] ITENE Research Center, Industrial Biotechnology Area, C/Albert Einstein 1, Paterna, 46980 Valencia, Spain; ja.tamayoramos@gmail.com

[5] UNLOCK Large Scale Infrastructure for Microbial Communities, Wageningen University and & Research and Delft University of Technology, 6708 WE Wageningen, The Netherlands

* Correspondence: thanaporn.lao@biotec.or.th (T.L.); peter.schaap@wur.nl (P.J.S.)

Dataset S1: *A. niger* AQPs sequences and domain identification

- **AQP Conserved structural features.** Loop B and E contain conserved motifs important for AQP classification. Picture adapted from Kruse et al., 2006.

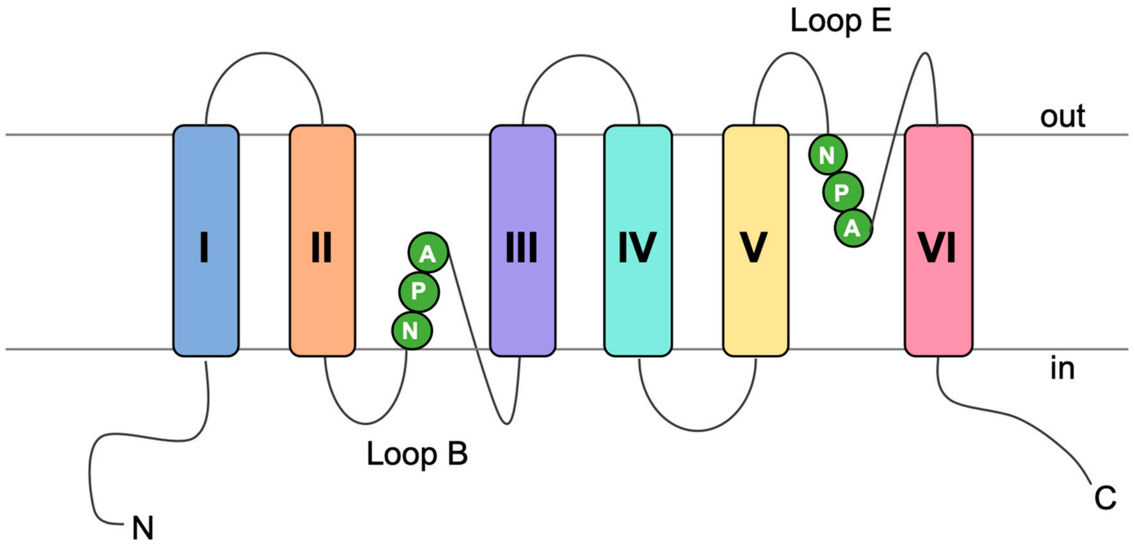

- **AQP amino acid sequence annotation**
  - Transmembrane domains I to VI are coloured as indicated above.
  - Cysteine residues are indicated in bold.
  - NPA motifs in loop B and E are indicated in green and bold.
  - Conserved sequences in loop B and E are underlined.

>**AQPA**_ATCC64974_2160 (5 transmembrane domains)
MGPQQWFRKRNPDSQTAPMYRSSNNQLPMLHLADTTRNNFIAAVGEFVGTFLFLFFSFAGTQVSNTPKPAPGSPP
NTSNLLYSSLCFGFSLMVNVWAFYRVTGGLFNPAVTLALCLVGGLSPIRGVILFGVQLIAGIASAGVVGALFPGD
LNVGTRLGGGASISQGLFIEVFLTAQLVFIIIMLAVVKHKSTFLAPVGIGLVFFVTEMVGDYYTGGSLNPARSLG
PDVINRSFPGYHWIYWVGPLLGSLLACGFFTFLKMFQYTTVNPGQDYNE

>**AQPB**_ATCC64974_25380
MVHPVRSLNKELDQVDTESRGYRSSDTTEGGSSKVEDGDEPIEETSRRGVSRNKSHGTQRTNFSRNKSHASHRTG
VSQNQSYRSQPPATLRNQSYGSQAQFSLAGPNAYYGPLPQQGAYMHPEYQSFNPQYGNMQGDKPVWSLAQPLPHV
VRDGMRYGALPEDRKEERDEDGRERPPPASEEPPTDIPKTDEARENGEEPQNEMGFFNKWSKIRYYLREPLGEWL
GTTIAITLGLCGGLSTYTSDGQAGSWMTQSACWGFSFMIGIYIVGGISGGHLNPAITISMSLWRGFPARRCVIYI
LAQLIGAITAGGFAYAIYHDAIVNLSVETNLPQSETTASQAFLTLPKQFVSPATAFFNEFLGTAILVGTIMALGD
DTNAPPGAGMQAFIIGILITVLVLALGYNTGGAFNGPRDFGPRLVAVMAGWGGHLFKEYHAWWIWGPWVADIFGG
LFGAFIYDLVVFTGGESPINYPPRRRKRALLIKEKNLRSKLRFGRRKIGDIERAVEENQD

>**AQPC**_ATCC64974_26080 (5 transmembrane domains)
MAGLLFRPQNDIDPSVNVQQLSCKPHVQPFVGRIGGNQGIVLDRADPDNAEYLRKVPDAAPLMSARDAFNVRGFT
DLDLWRFAVVECVGTMMLAFITAWAAATPANVAPPSPSTPAGIFATTAFLGPLVGAVTNWLLLTLFIFSFSSVSG
SHLNPTITLATFFARLISLPRMVLYLCGQILGGALAGWMLQSAFGSGQYSVGGCVVDTALVPVREAFVLEFICSL
TLIFLSFGVGLDPRQVRVYGAALSPWLVGMVLGAVSLGSAYTREGYGGACVLFFYTLTSKCSVPSECPEANFRFQ
R

>**AQPD**_ATCC64974_52260
MFLNTGLSRKFLKPYVAEFLGTALLIVIGDGVVAQCLLSDYQYGTWLSINIAWAAAVCISGYLSDPGPTINPAVT
ICMALVRPTPGQWRKLPGKLFAQFLGGFVGAAIVYINYRSAIKDWDPEFTIPGGSILSPRGHHSAGIFSTYPAAF
FESNWEAAFSELLGSAVLMFGILSISDPVNAVRFHSPQVTVFFLLTAIGAALGWQTGYAINPARDFGPRLFSAFI
YGREVFTAANYYFVVPIFAPIVGCVVGAATYDTFLYEGDGSRITDALDNVEDRDGALRLH

>**AQPE**_ATCC64974_71680
MDDGETRRPRATSDAISPRGENPPTSPLSPTRGRRGTAGDADFVSPTRRVSGRDGSGAGVRRRSSAASRRSGGSG
IPSLAPRTTLAGRTQGRFTMAGPEETPQLRLAHEPFVQPGYGDLNPSYEQPANSKPVWGLAKPLPRVVRPGMVPT
KEELLEARQNIQLPAENSQKLGLEVDPNDLELGQIEKTADPRKMAAQVEDARIQRENNFMNKILSGDATTTRQGS
RLSRTSSSRIRRPSAWDLPPENLSTVPEGETPAPSETHEEPPQMSSEEPLEPVLEEPELRADDGKDGMMDDLPNL
EEIEAAYPEDLHPLVQELVEEEVHNNHTTWSVIRTHHREALAESLGVFVQLFVGFCGDLAVTVANAGNPNTTDWV
WGFATMMAIYVSGGVSGAHLNPTITIMLWFYRGFPKSKMPEYFAAQFLGAFIAALAAYGLYYHSIQHYLLTNSTT
GIITSFVTSQRETWIGPGTAFFTEFLGTMILTVVVLALGDDQNAPPGAGMNSLIVGLMVTCNTMSFAYQTGAALN
PSRDFGPRLALLALGYGSSLFTNPYWFYGPWAGSLAGSFLGAFLYDFMIFTGGESPVNYPWERTQRAMRKSRMKW
GKRLHLSRRDRGEKTVR

>**AQPF**_ATCC64974_75350
MVVTYLPEYESDETHPQHHGLEPAIPPFAGRMGGNQDFVVDRTDPKNSKVLERVPDAAPCMTLKEIFDLRGFLSV
DLWKFAVLECIASMMNVFITCWVTTHPLSATTSPKGQAGVYGTVTFFSPTFGGLTNLLLTPLLIYTFSPSSGGHI
SPTITLATFFARIITFPRMILYLAGQTLGGALAGFAMHSAYGTREFTVGGCHIDTTMVSAKDGLVIEFFACLILI
FLAFGVALDPRQAKVFGHAVSPWLVGVVLGIVTWGTAFTREGYIGASVNPARCFGAYVASDFPTYHWIHWVGPLA
AAVAHGLVYFVDPLWKDPRAE

>**AQPG**_ATCC64974_111300 (5 transmembrane domains)
MIPETEKSGCREKPAHGIAPFPQTHPDGYQKTVKTDTEMPVWKCLASIDMWRCTILECLASMMYTFMLTWVTIAP
TSKDRDDPGNLSYYSLNNYDWVRFLPTSIVNVCLLPLIIFTFARSTGGYVNPSITFAAYFLRRISLSRAVMYIGG
QVIGIVLAVWAVQEAYGSTGFDIGCIISRGGVSIRNAYIAEVVSGFAMVVTVMYIAAGRKSGVLFGDAMSPWMVG
IATEAAFWLSRLIWQGHPGASRQLI

- **AQPs amino acid sequences used in classification**

Accession number	Species	Predicted class
SPB42568.1 (AQPA)	*A. niger* ATCC64974	orthodox-AQP
SPB44895.1 (AQPB)	*A. niger* ATCC64974	Yfl054-like-AQGP
SPB44966.1 (AQPC)	*A. niger* ATCC64974	XIP
SPB47598.1 (AQPD)	*A. niger* ATCC64974	**γ**-AQGP
SPB49549.1 (AQPE)	*A. niger* ATCC64974	Yfl054-like-AQGP
SPB49917.1 (AQPF)	*A. niger* ATCC64974	XIP
SPB53530.1 (AQPG)	*A. niger* ATCC64974	-
CBF75100	*A. nidulans*	α-AQGP
EAW06528	*A. clavatus*	α-AQGP
EED48588	*A. flavus*	α-AQGP
BAE57020	*A. oryzae*	α-AQGP
EGU81740	*F. oxysporum*	α-AQGP
JQ585594	*L. bicolor*	β-AQGP
EFQ30640	*C. graminicola*	β-AQGP
GAA93674	*M. osmundae*	β-AQGP
XP658434	*A. nidulans*	**γ**-AQGP
EED45967	*A. flavus*	**γ**-AQGP
XP001400456	*A. niger* CBS513.88	**γ**-AQGP
GAA84320	*A. kawachii*	**γ**-AQGP
CAA38096	*S. cerevisiae*	FPS1-like-AQGP
XP453974	*K. lactis*	FPS1-like-AQGP
XP445318	*C. glabrata*	FPS1-like-AQGP
GAA2303	*S. cerevisiae*	Yfl054-like-AQGP
XP680887	*A. nidulans*	Yfl054-like-AQGP
EED52139	*A. flavus*	Yfl054-like-AQGP
XP001825721	*A. oryzae*	Yfl054-like-AQGP
CAK42703	*A. niger* CBS513.88	Yfl054-like-AQGP
XP001217233	*A. terreus*	Yfl054-like-AQGP

EGU87897	*F. oxysporum*	Yfl054-like-AQGP
GAA86900	*A. kawachii*	Yfl054-like-AQGP
EAW09088	*A. clavatus*	Yfl054-like-AQGP
EJP62256	*B. bassiana*	δ-AQGP
ELQ43205	*M. oryzae*	δ-AQGP
AFJ15556	*L. bicolor*	δ-AQGP
AGE95845	*E. cuniculi*	SIP-like
EEQ81370	*N. ceranae*	SIP-like
EIJ87245	*N. parisii*	SIP-like
XP001547129	*B. cinerea*	orthodox-AQP
ADC55259	*S. cerevisiae*	orthodox-AQP
EGU79346	*F. oxysporum*	orthodox-AQP
EAW13465	*A. clavatus*	orthodox-AQP
CBF7893	*A. nidulans*	orthodox-AQP
EED52312	*A. flavus*	orthodox-AQP
XP001211324	*A. terreus*	orthodox-AQP
EED52460	*A. flavus*	orthodox-AQP
XP001825978	*A. oryzae*	orthodox-AQP
XP001390456	*A. niger* CBS513.88	XIP
EED45369	*A. flavus*	XIP
XP00139648	*A. niger* CBS513.88	XIP
EGX52763	*A. oligospora*	XIP
EGU75229	*F. oxysporum*	XIP
