# Supplementary material for: Identification of Aspergillus niger Aquaporins Involved in Hydrogen Peroxide Signaling
Source: J Fungi (Basel). 2023 Apr 21;9(4):499. doi: 10.3390/jof9040499 (PMC10144872; doi:10.3390/jof9040499)
Supplement: Supplementary file 1 [file jof-09-00499-s001.zip › Supplementary Figure S1_Plasmid construction.pdf]

Identification of *Aspergillus niger* aquaporins involved in hydrogen peroxide signaling

Thanaporn Laothanachareon [1,2,*], Enrique Asin-Garcia [1,3], Rita J.M. Volkers [1], Juan Antonio Tamayo-Ramos [4], Vitor A.P. Martins dos Santos [3] and Peter J. Schaap [1,5,*]

[1] Laboratory of Systems and Synthetic Biology, Wageningen University & Research, 6708 WE Wageningen, The Netherlands; enrique.asingarcia@wur.nl (E.A.-G.); j.m.volkers@nvwa.nl (R.J.V.)

[2] Enzyme Technology Laboratory, Biorefinery and Bioproduct Technology Research Group, National Center for Genetic Engineering and Biotechnology, 113 Thailand Science Park, Khlong Luang, Pathumthani 12120, Thailand

[3] Biomanufacturing and Digital Twins, Wageningen University and Research, 6708 PB Wageningen, The Netherlands; vitor.martinsdossantos@wur.nl

[4] ITENE Research Center, Industrial Biotechnology Area, C/Albert Einstein 1, Paterna, 46980 Valencia, Spain; ja.tamayoramos@gmail.com

[5] UNLOCK Large Scale Infrastructure for Microbial Communities, Wageningen University and & Research and Delft University of Technology, 6708 WE Wageningen, The Netherlands

* Correspondence: thanaporn.lao@biotec.or.th (T.L.); peter.schaap@wur.nl (P.J.S.)

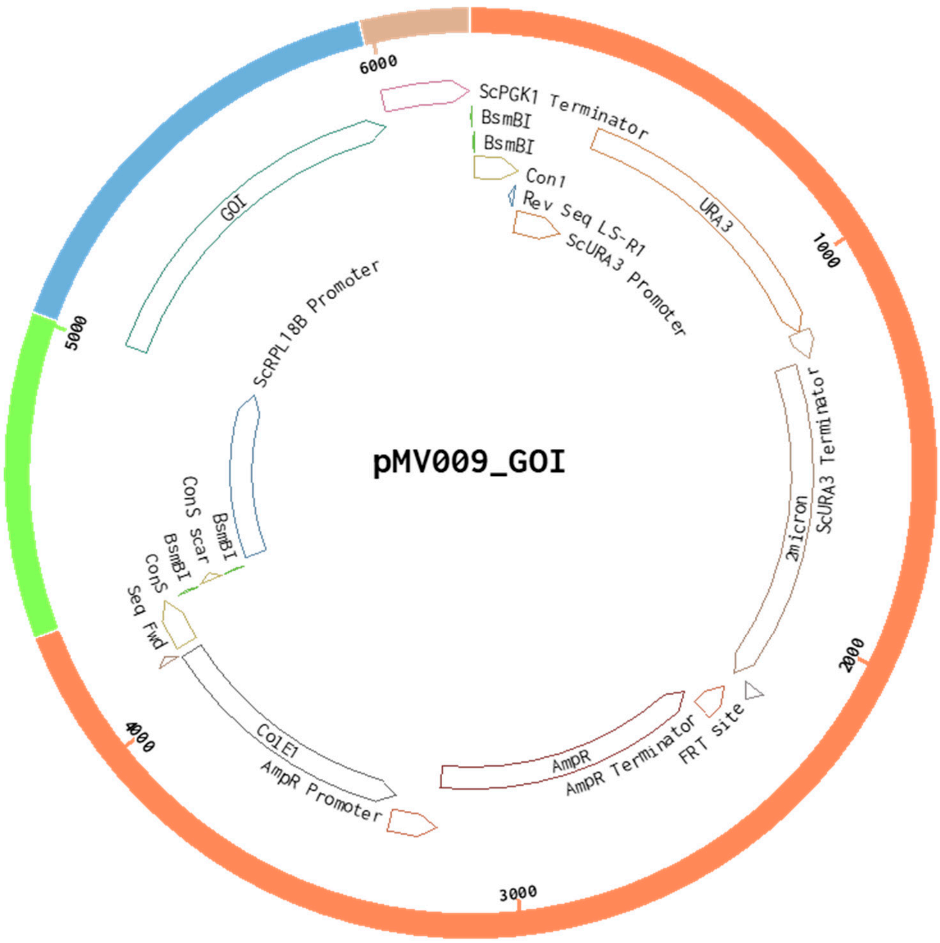

Figure S1: Plasmid containing *A. niger* AQPs. Each *A. niger* AQP was inserted in between pRPL18B and tPGK1 terminator and then cloned into high copy number plasmid pMV009. The plasmid contains *AmpR* gene as an ampicillin resistance gene and *URA3* gene as a marker gene. Once the AQPs were ligated into the plasmid, GFP sequence was removed.
