# Supplementary material for: Identification of Aspergillus niger Aquaporins Involved in Hydrogen Peroxide Signaling
Source: J Fungi (Basel). 2023 Apr 21;9(4):499. doi: 10.3390/jof9040499 (PMC10144872; doi:10.3390/jof9040499)
Supplement: Supplementary file 1 [file jof-09-00499-s001.zip › Supplementary Table S1_Primers.pdf]

Identification of *Aspergillus niger* aquaporins involved in hydrogen peroxide signaling

Thanaporn Laothanachareon [1,2,*], Enrique Asin-Garcia [1,3], Rita J.M. Volkers [1], Juan Antonio Tamayo-Ramos [4], Vitor A.P. Martins dos Santos [3] and Peter J. Schaap [1,5,*]

[1] Laboratory of Systems and Synthetic Biology, Wageningen University & Research, 6708 WE Wageningen, The Netherlands; enrique.asingarcia@wur.nl (E.A.-G.); j.m.volkers@nvwa.nl (R.J.V.)

[2] Enzyme Technology Laboratory, Biorefinery and Bioproduct Technology Research Group, National Center for Genetic Engineering and Biotechnology, 113 Thailand Science Park, Khlong Luang, Pathumthani 12120, Thailand

[3] Biomanufacturing and Digital Twins, Wageningen University and Research, 6708 PB Wageningen, The Netherlands; vitor.martinsdossantos@wur.nl

[4] ITENE Research Center, Industrial Biotechnology Area, C/Albert Einstein 1, Paterna, 46980 Valencia, Spain; ja.tamayoramos@gmail.com

[5] UNLOCK Large Scale Infrastructure for Microbial Communities, Wageningen University and & Research and Delft University of Technology, 6708 WE Wageningen, The Netherlands

* Correspondence: thanaporn.lao@biotec.or.th (T.L.); peter.schaap@wur.nl (P.J.S.)

Table S1: Primers used in this study

A: Primers for yeast experiment

Primer	Sequence 5´ → 3´
SC_aqpA.1_FW	GCAT**CGTCTC**ATC**GGTCTC**ATATGGGGCCGCAACAATGGTTTC
SC_aqpA.1_RV	ATGC**CGTCTC**CGTGACCCGGGCGCTGGCTTGG
SC_aqpA.2_FW	GCAT**CGTCTC**ATCACCTCCAAACACCTCAAACCTG
SC_aqpA.2_RV	ATGC**CGTCTC**A**GGTCTC**AGGATCCCACCTGCTCTGCCCCGTG
SC_aqpB.1_FW	GCAT**CGTCTC**ATC**GGTCTC**ATATGGTGCACCCGGTTAGATCTCTC
SC_aqpB.1_RV	ATGC**CGTCTC**CGGCGCCCCTCCTGGAGGTTTCTTC
SC_aqpB.2_FW	GCAT**CGTCTC**ACGCCTCTCGCAACAAAAGTCATGGAAC
SC_aqpB.2_RV	ATGC**CGTCTC**CTCCCTTTCTTCCTTTCGATCCTCCGGCAAGGCTCCATACCGCATTCCGTCCCGGAC
SC_aqpB.3_FW	GCAT**CGTCTC**AGGGACGAGGATGGACGAGAACGCC
SC_aqpB.3_RV	ATGC**CGTCTC**CGGCCGAGAGTGATGGCGATGG
SC_aqpB.4_FW	GCAT**CGTCTC**AGGCCTCTGCGGCGGGTTGTCAACC
SC_aqpB.4_RV	ATGC**CGTCTC**CTTTCGACGGAGAGGTTGACGATG
SC_aqpB.5_FW	GCAT**CGTCTC**AGAAACCAACCTCCCACAAAGCGAAAC
SC_aqpB.5_RV	ATGC**CGTCTC**CCCTCCTCGGCGGGTAATTGATCG
SC_aqpB.6_FW	GCAT**CGTCTC**AGAGGCGCAAGCGCGCACTGCTGATC
SC_aqpB.6_RV	ATGC**CGTCTC**C**GGTCTC**AGGATCCATCCTGGTTCTCCTCAACTG
SC_aqpC_FW	GCAT**CGTCTC**ATC**GGTCTC**ATATGGCAGGCCTACTGTTTCGTC
SC_aqpC_RV	ATGC**CGTCTC**A**GGTCTC**AGGATCCTGCCTTCCATGGCGGCAC
SC_aqpD.1_FW	GCAT**CGTCTC**ATC**GGTCTC**ATATGTTTCTCAACACTGGCCTCTC
SC_aqpD.1_RV	ATGC**CGTCTC**CTCCCCCTCATAGAGGAATGTGTCG
SC_aqpD.2_FW	GCAT**CGTCTC**AGGGACGGGTCGCGCATCACCGATG
SC_aqpD.2_RV	ATGC**CGTCTC**A**GGTCTC**AGGATCCGTGCAGCCGCAGAGCAC
SC_aqpE.1_FW	GCAT**CGTCTC**ATC**GGTCTC**ATATGGACGACGGGGAAACCAGG
SC_aqpE.1_RV	ATGC**CGTCTC**CAACGCGGCGGGTGGAGAAAC

Primer	Sequence 5′ → 3′
SC_aqpE.2_FW	GCAT**CGTCTC**ACGTTTCTGGCCGTGATGGCTCCG
SC_aqpE.2_RV	ATGC**CGTCTC**CTGTCTCTTCTGGGCCAGCCATGGTG
SC_aqpE.3_FW	GCAT**CGTCTC**AGACACCTCAGCTGCGCTTGGC
SC_aqpE.3_RV	ATGC**CGTCTC**CTTTCACTGGGAGCCGGGGTTTCGCC
SC_aqpE.4_FW	GCAT**CGTCTC**AGAAACCCATGAGGAACCCCCTCAG
SC_aqpE.4_RV	ATGC**CGTCTC**CAACGTAGATGGCCATCATGGTAGC
SC_aqpE.5_G-Block	GCAT**CGTCTC**ACGTTTCCGGTGGTGTGTCCGGTGCACATCTCAATCCG ACCATCACCATTATGCTATGGTTTTACCGCGGTTTCCCCAAGAGCAAGA TGCCCGAGTACTTCGCCGCGCAATTCTTGGGGGCCTTCATCGCCGCGCT CGCCGCGTACGGTCTTTACTATCACTCCATCCAACACTATCTCCTGACC AACTCCACCACGGGCATCATCACCAGCTTTGTGACAAGTCAGCGCGAA ACCTGGATCGGTCCTGGTACTGCCTTCTTTACCGAATTTCTGGGCACGA TGATACTGACGGTGGTGGTCCTGGCGCTGGGTGACGATCAGAACGCAC CCCCGGGAGCGGGTATGAACTCACTCATCGTGGGGCGGAGACGGCAT
SC_aqpE.6_FW	GCAT**CGTCTC**AGGGCTCATGGTGACCTGCAACACG
SC_aqpE.6_RV	ATGC**CGTCTC**A**GGTCTC**AGGATCCACGAACAGTCTTCTCTCCC
SC_aqpF_FW	GCAT**CGTCTC**ATC**GGTCTC**ATATGGTGGTCACTTATCTCCCCG
SC_aqpF_RV	ATGC**CGTCTC**A**GGTCTC**AGGATCCCTCCGCGCGAGGATCCTTC
SC_aqpG.1_FW	GCAT**CGTCTC**ATC**GGTCTC**ATATGATACCTGAGACAGAGAAGAGT
SC_aqpG.1_RV	ATGC**CGTCTC**CCATCTCGATCTTTGGAGGTGGGTGC
SC_aqpG.2_FW	GCAT**CGTCTC**AGATGACCCCGGTAACCTTTCCTAC
SC_aqpG.2_RV	ATGC**CGTCTC**A**GGTCTC**AGGATCCACTCAACGAAGCTGCCCC
SC_FPS1_FW	GCAT**CGTCTC**ATC**GGTCTC**ATATGAGTAATCCTCAAAAAGCTCTAAAC
SC_FPS1_RV	ATGC**CGTCTC**A**GGTCTC**AGGATCCTGTTACCTTCTTAGCATTACC
SC_pYTK001_FW	GCCTATGGAAAAACGCCAGC
SC_pYTK001_RV	CGCTTGGACTCCTGTTGATAG

Remark:

1. SC: primers for *Saccharomyces cerevisiae* experiment
2. underline letter: type specific overhangs
3. bold letter: restriction site for *BsaI* (GGTCTC) and *BsmBI* (CGTCTC)

B: Primers for RT-qPCR

Primer	Sequence 5´ → 3´
AN_qPCR_hist_FW	ACAATGACTGGCCGTGGAAAGG
AN_qPCR_hist_RV	ATACGCTTGACACCACCACGAC
AN_qPCR_aqpA_FW	CGCTATGCTTTGGGTTCTCG
AN_qPCR_aqpA_RV	CCAGACACAGGGCCAGAGTGACCG
AN_qPCR_aqpB_FW	CCACGGATATTCCGAAGACGGATG
AN_qPCR_aqpB_RV	AGAGTGATGGCGATGGTTGTCC
AN_qPCR_aqpC_FW	CGGTGGTGGAATGCGTTGGAACC
AN_qPCR_aqpC_RV	GGAGAGGGGGGAGCCACATTCG
AN_qPCR_aqpD_FW	ACTCCCGGACAATGGCGTAAAC
AN_qPCR_aqpD_RV	ATGGCAGCACCAACAAATCCAC
AN_qPCR_aqpE_FW	TGGTGAATCGCCGGTCAACTAC
AN_qPCR_aqpE_RV	TCATCCGACTCTTTCGCATCGC
AN_qPCR_aqpF_FW	TGACAGGACGGACCCTAAGAAC
AN_qPCR_aqpF_RV	GCTATGCATTCTAGCACTGCGAAC
AN_qPCR_aqpG_FW	ATCCCGGTGCAAGATCTAATG
AN_qPCR_aqpG_RV	CGAGACTACCATCCTCATCAAAG
AN_qPCR_goxC_FW	AATGCTGCCCGTGTGTATGGTG
AN_qPCR_goxC_RV	TGGGACGACATTTGCGTAGGAG
AN_qPCR_catR_FW	GCTGAAGTTGAACAGGCTGGTTTC
AN_qPCR_catR_RV	TCGGTGAAGTCAATGCCAGGAAC
AN_qPCR_lctA_FW	CGGAAATTGCCATCTCACC
AN_qPCR_lctA_RV	GGCGATGGAATCGTTGTTG

Remark:

AN: primer for *A. niger* experiments

C: Primers for *A. niger* experiment

Primer	Sequence 5´ → 3´
AN_aqpD_FW	ATGTTTCTCAACACTGGCCTCTCG
AN_aqpD_RV	TTAGTGCAGCCGCAGAGCACCG
AN_aqpD_KO_p1	GGAGTCCCTCTACCTTTCCAGG
AN_aqpD_KO_p2	CAATTCCAGCAGCGGCTTGGGAAGATTCGTAGGGGTACCG
AN_aqpD_KO_p3	ACACGGCACAATTATCCATCGCGATACTGGCTGGAATAGAGG
AN_aqpD_KO_p4	CGCCAAGAACAGTGTGTCC
AN_aqpD_KO_p11	GGTGCCGAGTCCGTTCGCC
AN_aqpD_KO_p12	CCAACCTTACCAAAGAATTCC
AN_aqpE_FW	ATGGACGACGGGGAGACCAGG
AN_aqpE_RV	TCAACGAACAGTCTTCTCTCCC
AN_aqpE_KO_p1	GGAGCAAGAATCAAGGAACG
AN_aqpE_KO_p2	CAATTCCAGCAGCGGCTTGGGCGAAGGGTGATAAAATTGCTCC
AN_aqpE_KO_p3	ACACGGCACAATTATCCATCGCCAGATTGTTTGAGAATGCC
AN_aqpE_KO_p4	GCGGCAAAGTTAGGCGACTCC
AN_aqpE_KO_p11	CCTCGGAAGACACAATGGATCG
AN_aqpE_KO_p12	CGAATGCGAGCAGCGAAACAGGG
AN_aqpF_FW	ATGGTGGTCACTTATCTCCCCG
AN_aqpF_RV	TTACTCCGCGCGAGGATCCTTCC
AN_aqpF_KO_p1	CGCTAAGATCGCTGGCACGCG
AN_aqpF_KO_p2	CAATTCCAGCAGCGGCTTGGGGCTGCTATCAAGATAAGTG
AN_aqpF_KO_p3	ACACGGCACAATTATCCATCGCATGGGAACGGAGTGTCGG
AN_aqpF_KO_p4	GGCTGCAGATAAGCTGGACG
AN_aqpF_KO_p11	CCGAAAGTATTTACAGTTGAC
AN_aqpF_KO_p12	GCGCTCTGATGTCTGGTCGG
AN_AOpyrG12FW_p5	AAGCCGCTGCTGGAATTG
AN_AOpyrG13RV_p6	CGATGGATAATTGTGCCGTGT

Primer	Sequence 5´ → 3´
AN_AOpyrG14FW_p7	ATTGACCTACAGCGCACGC
AN_AOpyrG15RV_p8	CCGGTAGCCAAAGATCCCTT
AN_AOpyrGintFW_p14	GGCAATTGTCGTGCAGTTTG
AN_AOpyrGintRV_p13	AATTCCACGGCCAGCAATAA

Remark:

underline letter: overlapped sequences of *pyrG* gene
